# Pharmacokinetic-Pharmacodynamic Modeling to Study the Antipyretic Effect of Qingkailing Injection on Pyrexia Model Rats

**DOI:** 10.3390/molecules21030317

**Published:** 2016-03-07

**Authors:** Zhixin Zhang, Lingling Qin, Long Peng, Qingqing Zhang, Qing Wang, Zhiwei Lu, Yuelin Song, Xiaoyan Gao

**Affiliations:** 1 School of Chinese Pharmacy, Beijing University of Chinese Medicine, South of Wangjing Middle Ring Road, Chaoyang District, Beijing 100102, China; zzxzxz@163.com (Z.Z.); lingling19900220@126.com (L.Q.); penglong0829@163.com (L.P.); janezh1987@sina.com (Q.Z.); shadow152324@126.com (Q.W.); bucm1989@163.com (Z.L.); 2 Modern Research Center for Traditional Chinese Medicine, Beijing University of Chinese Medicine, No. 11 North Third Ring Road, Chaoyang District, Beijing 100029, China; syltwc2005@163.com

**Keywords:** pharmacokinetic-pharmacodynamic model, antipyretic effect, Qingkailing injection, PK marker

## Abstract

Qingkailing injection (QKLI) is a modern Chinese medicine preparation derived from a well-known classical formulation, An-Gong-Niu-Huang Wan. Although the clinical efficacy of QKLI has been well defined, its severe adverse drug reactions (ADRs) were extensively increased. Through thorough attempts to reduce ADR rates, it was realized that the effect-based rational use plays the key role in clinical practices. Hence, the pharmacokinetic-pharmacodynamic (PK-PD) model was introduced in the present study, aiming to link the pharmacokinetic profiles with the therapeutic outcomes of QKLI, and subsequently to provide valuable guidelines for the rational use of QKLI in clinical settings. The PK properties of the six dominant ingredients in QKLI were compared between the normal treated group (NTG) and the pyrexia model group (MTG). Rectal temperatures were measured in parallel with blood sampling for NTG, MTG, model control group (MCG), and normal control group (NCG). Baicalin and geniposide exhibited appropriate PK parameters, and were selected as the PK markers to map the antipyretic effect of QKLI. Then, a PK-PD model was constructed upon the bacalin and geniposide plasma concentrations *vs.* the rectal temperature variation values, by a two-compartment PK model with a Sigmoid E_max_ PD model to explain the time delay between the drug plasma concentration of PK markers and the antipyretic effect after a single dose administration of QKLI. The findings obtained would provide fundamental information to propose a more reasonable dosage regimen and improve the level of individualized drug therapy in clinical settings.

## 1. Introduction

Qingkailing injection (QKLI), a modern Chinese medicine preparation derived from An-Gong-Niu-Huang Wan, is prepared from cholic acid, hyodeoxycholic acid, baicalin, and five medicinal materials, namely *Gardeniae* *Fructus* (Zhizi), *Bubali Cornu* (Shuiniujiao), *Margaritifera Concha* (Zhenzhumu), *Isatidis Radix* (Banlangen), and *Lonicerae* *Japonicae Flos* (Jinyinhua). A wide pharmacological spectrum has been revealed for this famous injection, such as antipyretic effect, anti-inflammation, and vasodilatation, *etc*. [1]. QKLI has been widely used for the treatment of high fever in clinical settings; however, no study is available concerning its dose-effect relationship up to now, and the clinical administration depends only on clinical experience [2]. The bulletins about warnings of the potential for severe adverse drug reactions (ADRs) to QKLI were posted by the Chinese National Adverse Drug Reaction Monitoring Center (CNADRMC) in November 2001 and April 2009, respectively. A significant number of ADR cases led the QKLI to be the second leading cause of ADRs in all traditional Chinese medicine injections. Aiming to find the possible allergens of QKLI, numerous studies have been performed to reveal the chemical material basis and to develop quality control techniques [3,4,5,6,7,8,9]; nonetheless, insufficient evidence could be provided by those studies. Hence, it is critical to characterize the dose-effect relationship of QKLI to subsequently guide the clinical use of this herbal medicine [10].

There are three main means to describe the dose-effect relationship by correlating the pharmacokinetic (PK) and pharmacodynamic (PD) data, including PK/PD integration, dose titration study, and PK-PD modeling. PK/PD integration is such a measurement to integrate *in vitro* PD profiles with PK parameters obtained from separated PK and PD studies, which has been widely used to study antimicrobial drugs nowadays [11]. In dose titration studies, at least three dose levels are required, and the effective outcomes are measured to connect with the corresponding dose directly [12,13,14]. However, it is difficult to obtain the optimal dose because there is no information regarding the relationship between plasma concentration and the PD dataset [15,16,17,18]. Moreover, the dose titration study with several dose groups suffers from a large consumption of animals and labor. By comparison, PK-PD modeling is a more effective, but less expensive, approach which is also a dynamic process to correlate concentration-time course and effect-time profile relationships. Since PK and PD datasets are obtained from the same individual, and the dose-effect relationship can be constructed from only one single dose administration trial, indicating that the PK-PD modeling offers both theoretical and practical advantages over PK/PD integration and dose titration studies. Toutain *et al.* determined the dosage regimen of nimesulide in dogs by both PK-PD modeling and classical dose titration study, and the results suggested that PK-PD modeling rather than dose-titration study could be achieved following a single dosing [19], in brief, to construct the effective plasma concentration-time curve, to estimate the PD parameters after administration of a single dose, and finally to establish the relationship between the plasma concentration and the effect. PK-PD modeling has been widely used in pre-clinical and clinical studies, which contribute to learning the process of efficacy that varies with time and plasma concentration more comprehensively and accurately, thus providing valuable preferences for optimizing the clinical dosage, improving the therapeutic efficacy and reducing the toxic and side effects [20,21,22,23,24].

Series of caffeoylquinic acids, in particular the primary ones, chlorogenic acid and neochlorogenic acid, from *Lonicerae Japonicae* *Flos* and *Gardeniae Fructus* are widely suspected to be the allergens that contribute to ADRs of QKLI [25,26,27]. In addition, baicalin, geniposide, cholic acid, and hyodeoxycholic acid were documented as the four index components for the quality control of QKLI in Chinese Pharmacopeia [2]. Therefore, these six constituents were chosen as pharmacokinetic marker candidates to make a correlation with the antipyretic effect of QKLI by PK-PD modeling. The findings obtained are expected to provide fundamental data for further study on population pharmacokinetics/pharmacodynamics (PPK/PPD), to subsequently construct optimal dosage regimen more reasonably and to improve the level of individualized drug therapy.

## 2. Results

### 2.1. Pharmacokinetic Profiles of Six Analytes in Normal Treated Group and Pyrexia Model Group Rats

**Figure 1:**
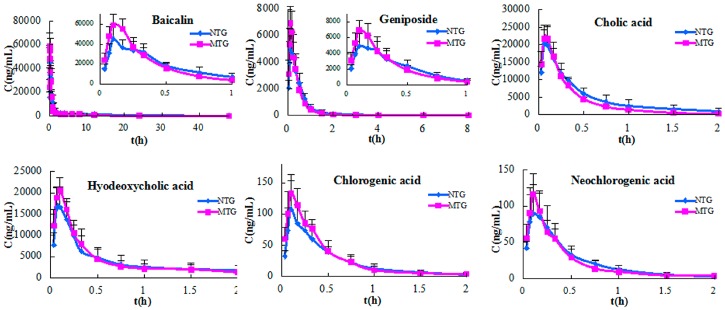
Plasma concentration-time profiles of six analytes in NTG and MTG rats after intraperitoneal administration of QKLI.

Pharmacokinetic behaviors of the six primary compounds in QKLI were compared between normal treated group (NTG) and pyrexia model group (MTG) rats. Figure 1 shows the mean (*n* = 8) concentration-time curves of the six analytes in the two groups after intraperitoneal administration of QKLI, and the main pharmacokinetic parameters are summarized in Table 1. In both groups, baicalin showed the slowest elimination (t_1/2_, 12.32 h and 10.34 h for NTG and MTG, respectively) and could be detected even after 48 h, and the t_1/2_ values of geniposide were calculated as 1.90 h and 1.93 h in NTG and MTG, respectively, whereas cholic acid (t_1/2_, 0.61 h and 0.56 h for NTG and MTG, respectively), hyodeoxycholic acid (t_1/2_, 0.64 h and 0.68 h for NTG and MTG, respectively), chlorogenic acid (t_1/2_, 0.32 h and 0.34 h for NTG and MTG, respectively), and neochlorogenic acid (t_1/2_, 0.24 h for both NTG and MTG) were eliminated rapidly. The statistical analysis results suggested that both C_max_ and AUC values (both AUC_0-t_ and AUC_0-∞_) of baicalin in MTG rats, as well as the C_max_ of geniposide in MTG rats, were significantly greater than those of NTG rats (*p* < 0.05), whereas no significant difference was found for the other parameters of baicalin and geniposide between the two groups. For the other four analytes, including cholic acid, hyodeoxycholic acid, chlorogenic acid, and neochlorogenic acid, there was no significant difference (*p* > 0.05) for all pharmacokinetic parameters between NTG and MTG.

### 2.2. Temperature Analysis

**Figure 2:**
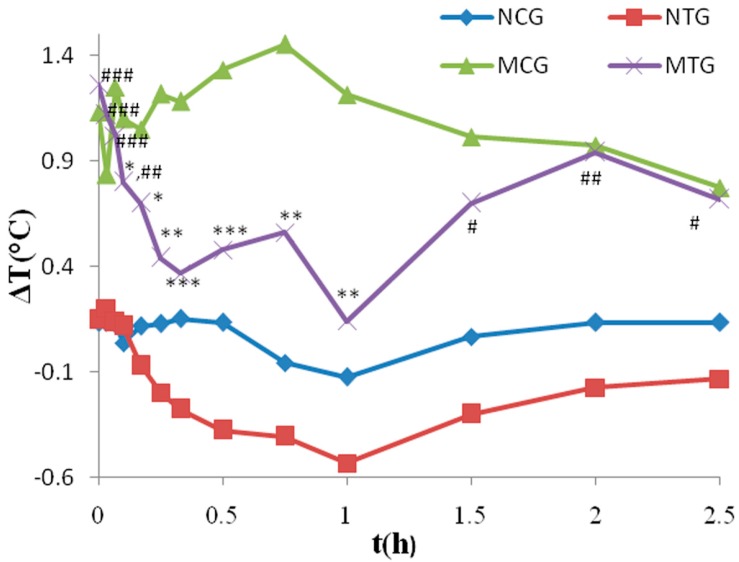
The mean time-course of rectal temperatures in four groups before and after intraperitoneally injected with QKLI or 0.9% saline. ∆T is the difference in rectal temperature from the basic value. * *p* < 0.05, ** *p* < 0.01, *** *p* < 0.001 compared with MCG rats. ^#^ *p* < 0.05, ^##^ *p* < 0.01, ^###^ *p* < 0.001 compared with NCG rats.

The PD indicators were calculated using the Equation (1). After subcutaneous injection of yeast suspension, the rectal temperature of model control group (MCG) rats decreased initially, and then increased rapidly to the maximum appreciation (1.38 ± 0.21 °C), which was significantly higher than the temperature change in normal control group (NCG) rats (*p* < 0.001), indicating that the pyrexia model was successfully established. The fever could last for almost 13 h in MCG rats. Figure 2 shows the mean (*n* = 8) change of rectal temperatures-time curves of all four groups from 0 to 2.5 h after intraperitoneal treatment with QKLI or vehicle. On the other side, the increment of body temperature in MTG rats was remarkably inhibited within 1 h after the administration of QKLI and the temperature reached the lowest value at the time point of 1 h. Significant differences occurred for the temperatures from 0.1 to 1 h between MTG and MCG rats (*p* < 0.05), yet no significant difference was occurred between MTG and NCG from 0.17 to 1 h. The rectal temperature change-time curve of MTG began to rise from the time point of 1 h, and there was no significant difference between MTG and MCG rats for the time points after 1.5 h. The rectal temperature change-time curve of NTG decreased slightly after injection of QKLI, but exhibited no significant difference compared with the NCG rats.

Above all, QKLI could not affect the normal rats, nonetheless, showed significant antipyretic effects on yeast-induced fever rats.

### 2.3. PK-PD Modeling

**Figure 3:**
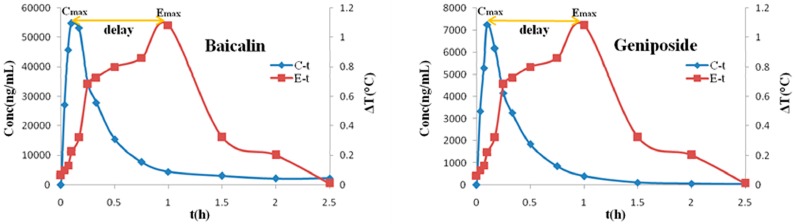
The peak effect lags behind the peak concentration of baicalin and geniposide after intraperitoneal administration of QKLI. ∆T is the absolute value of the change in rectal temperature from MCG and MTG.

**Figure 4:**
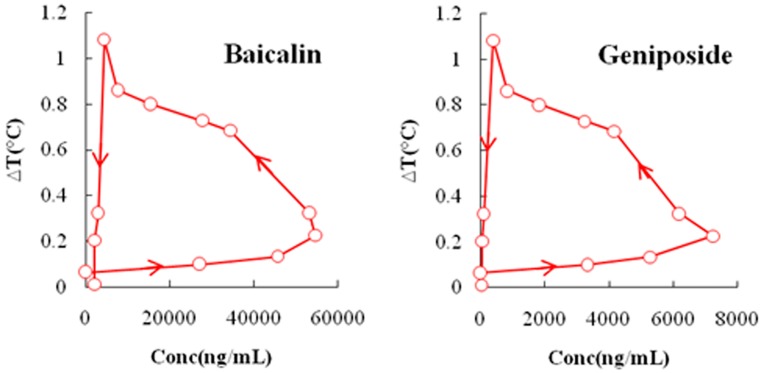
Anticlockwise hysteresis loop indicating equilibration delay between the effect (rectal temperature change) and the plasma concentrations of baicalin and geniposide.

**Figure 5:**
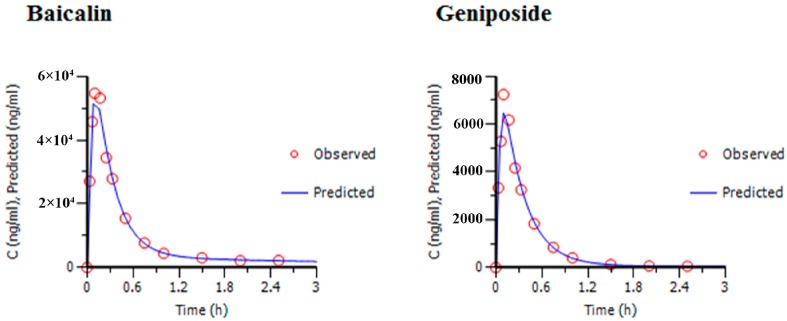
Prediction and observed mean plasma concentrations of baicalin and geniposide *vs*. time profiles for the two-compartment PK model.

**Figure 6:**
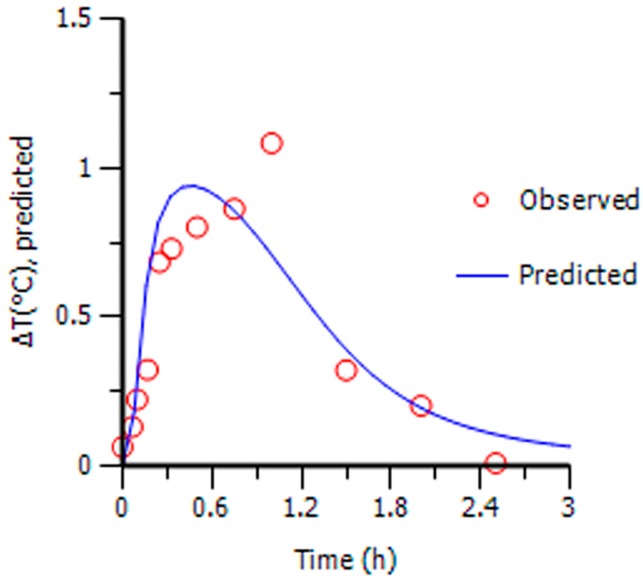
Prediction and observed mean antipyretic effect *vs.* time profiles for Sigmoid E_max_ PK-PD model.

Due to the effective duration of QKLI is quite short, PK and PD data obtained during 2.5 h after intraperitoneal injection was adopted to carry out PK-PD modelling. Table 2 shows the plasma concentration of baicalin and geniposide and the corresponding body temperature change of MTG rats during 2.5 h after QKLI administration. Figure 3 shows the mean plasma concentration-time curves (blue curves) of baicalin and geniposide and the corresponding time-courses of the body temperature change (red curves) of MTG rats. A significant hysteresis was observed with maximum effect (E_max_) occurring at 1 h while C_max_ of two markers achieved at 0.14 h, indicating that there was a delay in drug concentrations arriving the action site; thus, a PK-PD model with a separated effect compartment was introduced for the analysis of the dataset. The anti-clockwise hysteresis loops of baicalin and geniposide are showed in Figure 4, which highlights the delay between effect and plasmic distribution and indicates the existence of the effect compartment for both compounds. As showed in Figure 5 and Figure 6, both pharmacokinetic profiles of baicalin and geniposide in MTG rats were well fitted to the two-compartment model with a lag time, and best fitness with the Sigmoid E_max_ model was found to the PK-PD data. The parameters of baicalin and geniposide calculated using Ep2 were summarized in Table 3. The E_max_ of baicalin and geniposide were 1.31 °C and 1.23 °C, which were equal to 94.9% and 89.1% of the maximum temperature increment (1.38 °C) in MCG rats, respectively, indicating that these two compounds played as the determinant roles for antipyretic effect of the QKLI. The EC_50_/C_max_ value of baicalin and geniposide were calculated as 18.5% and 15.5%, respectively, suggesting that slight transport occurred for both constituents from central compartment to effect compartment. The γ values of baicalin and geniposide were determined as 2.03 and 2.08, respectively.

## 3. Discussion

In order to observe the full absorption process of all analytes, four time points (0.033, 0.067, 0.1, and 0.17 h) were included for blood sampling in the first ten minutes. The six constituents could be rapidly absorbed into the circulation system with T_max_ among 6~10 min after intraperitoneal administration of QKLI. Quick eliminations were observed for chlorogenic acid, neochlorogenic acid, cholic acid, and hyodeoxycholic acid (all t_1/2_ values lower than 0.7 h), especially chlorogenic acid and neochlorogenic acid, which were expelled quickly from circulation system and only determined as trace distribution (below the LLOQ) in plasma 2 h after administration.

Yeast-induced fever leads to an intense inflammatory reaction caused by fester at the injection site, which is the most widely used pathogenic fever model for antipyretic medicines. In accordance with previous observations, an initial temperature fall occurred 2 or 3 h post-injection with values among 0.5~1.5 °C, and then an increase of about 1.0 °C above basal temperature was observed 4 h later, and the fever usually lasted for 9~18 h [28,29,30,31,32]. In the present study, the NCG and MCG rats were sampled in parallel to make all datasets comparable. All the measured temperatures were put into Equation (1) to obtain the PD indicators, which were further adopted to construct PK-PD model.

It is reasonable to regard that the dense sampling was conducted for describing pharmacokinetic behaviors of six analytes more completely and investigating the relationship between plasma concentration and rectal temperature more thoroughly; thus, as many as 11 time points were set during the first 2 h following treatment, which was well matched with the short effect duration of QKLI. Meanwhile, in order to avoid the rats in exsanguine state, all rats were intragastricly administrated with saline regularly and the sampling volume collected every time was approximately 0.3 mL. Body temperature as the pharmacodynamic index of antipyretic medicines is characterized by objectivity, continuity, reliability, and repeatability; hence, rectal temperature, which could be measured expediently for rats, was chosen as the PD index in the current study.

As aforementioned, the EC_50_/C_max_ value of baicalin and geniposide were 18.5% and 15.5%, and the higher plasma protein binding rate of baicalin and geniposide were suspected to be responsible for this phenomen. The value of *γ* has clinical significance in relation to drug selectivity and effect sensitivity in the range of useful concentrations [33,34]. Generally speaking, when the drug has a low value of γ (γ < 1) *in vivo*, the PD profile should be relatively flat and with relatively small changes in effect over a wide range of drug concentrations; when γ = 1, the relationship is described with usual hyperbolic E_max_ model; when γ > 1, the curve becomes sigmoid with a steeper slope. Minor variations in concentration around EC_50_ can produce effects ranging from no effect to almost maximal effect; as γ increases to attain high values (γ > 5), the concentration range for a given effect diminishes to become a simple threshold. In the present study, the γ values of baicalin and geniposide were calculated as 2.03 and 2.08, respectively, which shows that the margin of safety is relatively narrow for QKLI [35].

A major advantage of PK-PD modeling is that the PD parameters can be estimated by investigation of a single dose administration trial, in contrast to classical dose titration studies which require multiple dosages to study the dose-effect relationship of a drug. Moreover, the PK and PD parameters can be used to evaluate the effectiveness by simulation of different dosage and different interval of administration for making an optimal dosage regimen [19]. In addition, the PK-PD approach offers the prospect of inter-species extrapolation. It is possible to estimate the dose for one species from an efficacious dose in another species with a well-defined equation when EC_50_ is independent of species and when the same AUC produces the same effect in two species [36]. Therefore, extrapolation from preclinical studies in animals, pyretic rats in present study, to clinical trials in human can be realized to provide references to design dosage schedules in clinics and to shorten the experimental period.

## 4. Materials and Methods

### 4.1. Materials

QKLI was purchased from YaBao Pharmaceutical Group Co., Ltd. (Batch No. 210905A, Beijing, China) and the yeast was supplied by Mauri Food Co., Ltd. (Hebei, China). The reference standards, namely baicalin, geniposide, cholic acid, hyodeoxycholic acid, chlorogenic acid, and neochlorogenic acid with a purity of over 98% for each, were purchased from the National Institute for the Food and Drug Control (Beijing, China). LC/MS-grade methanol and acetonitrile were supplied by Fisher Scientific (Fair Lawn, NJ, USA), formic acid of HPLC-grade was obtained from ROE Scientific (Newcastle, Delaware, DE, USA), and ultrapure water was prepared by the Synergy UV water purification system (Millipore Corp., Billerica, MA, USA).

### 4.2. Experimental Animals and Sample Collection

SPF-grade male Sprague-Dawley rats, weighing 180‒220 g, were supplied by Beijing Weitonglihua Laboratory Animal Technology Co., Ltd. (Beijing, China). All animal experiments were performed in accordance with the Guidelines for the Care and Use of Laboratory Animals, and the protocols were approved by the Animal Ethics Committee of the Beijing University of Chinese Medicine (Beijing, China). The rats were kept under controlled environmental conditions at a temperature of 20 ± 2 °C, a relative humidity of 60% ± 5%, a light/dark cycle of 12 h for one week before treatment. Standard chow and Milli-Q water were provided *ad libitum*. All rats fasted overnight but had free access to water prior to drug administration.

During the acclimation week, the rats’ rectal temperatures were monitored three times per day using a digital thermometer to expel the unqualified rats whose temperature variation was greater than 0.5 °C, and to accommodate rats to the thermometer stimulation. For each determination, feces of rat should be emptied firstly, the thermometer sonde with a dab of petroleum jelly was then inserted approximately 3 cm into the rat’s rectum, and the value was finally recorded after the reading stabilization.

Afterwards, thirty-two qualified rats were divided into four groups randomly, including the NCG, NTG, MCG, and MTG, respectively. All rats’ rectal temperatures were measured three times in the 30 min prior to the experiment and the average values were adopted as their respective basal body temperatures. At the outset of the experiment, the rats of MCG and MTG were given 20% aqueous suspension of yeast at a dose of 10 mL/kg by subcutaneous injection in the back of rats to establish the pyrexia model [6], while the NCG and NTG rats received an injection of equal volume of 0.9% saline. Five hours later, the rats of NTG and MTG were administrated with 5.2 mL/kg QKLI (equivalent to 23.04 mg/kg for baicalin; 2.38 mg/kg for geniposide; 9.88 mg/kg for cholic acid; 5.69 mg/kg for hyodeoxycholic acid; 0.04 mg/kg for chlorogenic acid; and 0.079 mg/kg for neochlorogenic acid) via intraperitoneal injection, whereas the MCG and NCG were given an equal volume of 0.9% saline in parallel. Serial blood sampling (0.3 mL) were performed at 0, 0.033, 0.067, 0.1, 0.17, 0.25, 0.33, 0.5, 0.75, 1, 1.5, 2.5, 4, 8, 12, 24, and 48 h after dosing from orbital venous plexus, and each blood was subjected into heparinized tube. The blood samples were centrifuged at 6000 rpm at 4 °C for 10 min, and the supernatants were transferred into another clean tube and stored at −80 °C until analysis. On the other side, the rectal temperatures of all rats were measured at 0.033, 0.067, 0.1, 0.17, 0.25, 0.33, 0.5, 0.75, 1, 1.5, 2, 2.5, 3, 4, 8, and 12 h following intraperitoneal injection.

### 4.3. Analytical Determination of Plasma Samples

The quantitative assays of baicalin, geniposide, cholic acid, hyodeoxycholic acid, chlorogenic acid, and neochlorogenic acid in rat plasma were performed by following the methodology previously raised in our laboratory, which was featured with satisfactory sensitivity, accuracy, precision, and successful application for the pharmacokinetic study of the six analytes in QKLI. Briefly, the plasma samples were pretreated by protein precipitation and separated by UPLC-MS/MS (Waters Corp., Milford, MA, USA) on a BEH C_18_ column (100 × 2.1 mm i.d., 1.7 μm; Waters Corp., Milford, MA, USA) using a mobile phase composed of 0.1% formic acid aqueous solution (A) and acetonitrile (B) with gradient elution [8]. Chromatographic separation was performed at 40 °C with a flow rate of 0.4 mL/min in 6 min. All analytes were monitored by multiple reaction monitoring (MRM) mode with negative electrospray ionization.

### 4.4. Pharmacokinetic Analysis

Pharmacokinetic parameters including maximum concentration (C_max_), time to reach C_max_ (T_max_), elimination half-life (t_1/2_), area under the curve (AUC), clearance (Cl) and mean residence time (MRT) of the six analytes in NTG and MTG were calculated by a non-compartment model using SPSS 17.0 (SPSS Inc., Chicago, IL, USA) and Phoenix WinNonlin Ver. 6.2.× and 6.3 (Pharsight Corporation, Mountain View, CA, USA), and the pharmacokinetic behaviors between the two groups were compared.

In addition, the compartment pharmacokinetic model was developed to highlight the pharmacokinetics of baicalin and geniposide in MTG rats. Goodness of fit of different compartmental model with or without a lag time was systematically compared using the Akaike Information Criterion (AIC).

### 4.5. PK-PD Simulation

The rats’ rectal temperatures measured by thermometer in MCG and MTG should be transformed into the temperature change data to facilitate PK/PD simulation, and the variation values were calculated with the following equation (Equation (1)):

(1) $$\Delta T_{t}=(T_{MTGt}-T_{MTG0})-(T_{MCGt}-T_{MCG0})$$

where ∆*T_t_* is the absolute change value in rectal temperature at time *t*, *T_MTGt_* is the rectal temperature of rat in MTG at time *t*, *T_MCGt_* is the rectal temperature of rat in MCG at the same time, *T_MTG_*_0_ is the basal temperature of rat in MTG, and *T_MCG_*_0_ is the basal temperature of rat in MCG. In addition, the differences in temperatures were contrasted among four groups.

The plasma concentrations of baicalin and geniposide and the rectal temperature change data in MTG rats were used to construct the relationship between the pharmacokinetics and pharmacodynamics. In comparison among different pharmacodynamic models such as the linear model and E_max_ model, sigmoid E_max_ model for PK-PD analysis were selected based on best fitness in terms of AIC using the following equation (Equation (2)):

(2) $$E=\frac{E_{\max}\times C^{\gamma }}{E{C_{50}}^{\gamma }+C^{\gamma }}$$

where E is the absolute change of rectal temperature and C is the concentration in the effect compartment; E_max_ is the maximal possible effect that describes efficacy; EC_50_, which describes the potency, is the concentration that produces 50% of the E_max_; and γ is the midpoint slop of the curve, a shape coefficient that describes the sensitivity of the concentration-effect relationship.

## 5. Conclusions

In comparison of the pharmacokinetic behaviors of six analytes between the NTG and MTG rats, baicalin and geniposide were selected as the pharmacokinetic markers to map the antipyretic effect of QKLI. Afterwards, the pharmacokinetic profiles of baicalin and geniposide and the change track of rectal temperature in MTG rats were used to construct the PK-PD model for the antipyretic effect of QKLI. The hysteresis between plasma concentration-time curve and the corresponding effect-time curve was observed and successfully explained by the introduction of effect compartment. PK-PD modeling of the antipyretic effect of QKLI was achieved by a two-compartment pharmacokinetic model with a Sigmoid E_max_ pharmacodynamic model. The constructed PK-PD model successfully estimated the efficacy of QKLI by investigation of a single dose administration trial, which would provide fundamental data for further study on PPK/PPD to make a more reasonable dosage regimen and to improve the level of individualized drug therapy.

## Figures and Tables

**Table 1:** Pharmacokinetic parameters of six analytes in NTG and MTG rats after intraperitoneal administration of QKLI.

PK Parameter	Baicalin		Geniposide		Cholic Acid		Hyodeoxycholic Acid		Chlorogenic Acid		Neochlorogenic Acid	
	NTG	MTG	NTG	MTG	NTG	MTG	NTG	MTG	NTG	MTG	NTG	MTG
**t_1/2_ (h)**	12.32 ± 0.99	10.34 ± 0.61	1.90 ± 0.83	1.93 ± 0.43	0.61 ± 0.12	0.56 ± 0.26	0.64 ± 0.11	0.68 ± 0.04	0.32 ± 0.10	0.34 ± 0.11	0.24 ± 0.06	0.24 ± 0.05
**t_max_ (h)**	0.112 ± 0.029	0.142 ± 0.038	0.123 ± 0.036	0.135 ± 0.038	0.074 ± 0.015	0.093 ± 0.015	0.089 ± 0.017	0.114 ± 0.031	0.147 ± 0.036	0.134 ± 0.071	0.135 ± 0.038	0.143 ± 0.087
**C_max_ (ng/mL)**	45,452.3 ± 14,647.02	59,063.86 ± 5387.80 *	5044.57 ± 1690.17	7083.28 ± 738.70 *	20,698.88 ± 5614.00	22,019.43 ± 3237.81	16,731.13 ± 4819.31	20,312.59 ± 2651.40	101.90 ± 42.26	125.70 ± 32.55	94.13 ± 40.16	110.58 ± 21.98
**AUC_0-t_ (h·ng/mL)**	51,016.23 ± 3566.65	67,832.84 ± 8114.55 *	2979.17 ± 581.50	2655.71 ± 536.63	10,038.96 ± 2720.33	8159.23 ± 1215.33	9297.71 ± 3099.90	9377.2 ± 2805.95	49.34 ± 13.19	53.93 ± 14.60	43.95 ± 12.40	44.54 ± 7.64
**AUC_0-∞_ (h·ng/mL)**	52,575.02 ± 3767.03	71,530.32 ± 8373.62 *	2999.47 ± 582.40	2670.90 ± 533.86	10,329.32 ± 2750.36	8426.33 ± 1324.79	9585.43 ± 3101.21	9789.65 ± 3114.40	51.08 ± 12.51	56.44 ± 15.86	44.58 ± 12.45	45.42 ± 7.96
**MRT_0-t_ (h)**	9.85 ± 0.83	8.22 ± 0.85	0.64 ± 0.17	0.47 ± 0.06	0.61 ± 0.19	0.48 ± 0.09	0.76 ± 0.18	0.68 ± 0.09	0.45 ± 0.10	0.40 ± 0.08	0.43 ± 0.09	0.38 ± 0.06
**MRT_0-∞_ (h)**	12.77 ± 1.00	9.84 ± 0.90	0.71 ± 0.17	0.53 ± 0.05	0.70 ± 0.22	0.57 ± 0.15	0.86 ± 0.20	0.80 ± 0.11	0.52 ± 0.14	0.47 ± 0.12	0.45 ± 0.11	0.41 ± 0.06

* *p* < 0.05, compared with NTG rats.

**Table 2:** The plasma concentration of baicalin and geniposide and the corresponding body temperature change of MTG rats during 2.5 h after QKLI administration.

t (h)	C (ng/mL)		∆*T* (°C)
	Baicalin	Geniposide	
0	0	0	0.065
0.033	23,053 ± 1533.71	3777.28 ± 1060.57	0.098
0.067	46,133.46 ± 3300.19	5616.80 ± 668.04	0.132
0.1	54,852.34 ± 5601.30	6921.36 ± 659.57	0.224
0.17	56,117.21 ± 8107.36	6684.34 ± 741.91	0.324
0.25	32,275.86 ± 7226.69	4248.63 ± 1139.85	0.684
0.33	19,552.27 ± 5468.90	3280.44 ± 799.09	0.729
0.5	13,953.74 ± 5684.01	1739.75 ± 560.55	0.801
0.75	6173.46 ± 2291.78	762.76 ± 404.31	0.862
1.0	3587.83 ± 929.05	322.06 ± 207.30	1.082
1.5	2536.16 ± 310.87	68.59 ± 23.30	0.382
2.0	2224.68 ± 206.74	42.08 ± 22.52	0.253
2.5	2050.67 ± 249.00	36.64 ± 19.13	0.081

**Table 3:** Predicted PD parameters of baicalin and geniposide for antipyretic effect of QKLI obtained from MTG rats.

Compound	E_max_ (°C)	EC_50_ (ng/mL)	γ	*k*_e0_ (1/h)
Baicalin	1.31	10,944.43	2.03	1.59
Geniposide	1.23	1094.33	2.08	1.35

*k*_e0_: rate constant between plasma and effect compartment.
